# In the presence of TGF-β1, Asperosaponin VI promotes human mesenchymal stem cell differentiation into nucleus pulposus like- cells

**DOI:** 10.1186/s12906-020-03169-y

**Published:** 2021-01-14

**Authors:** Yong-tao Niu, Lin Xie, Rong-Rong Deng, Xiao-yu Zhang

**Affiliations:** Department of Spine Surgery, Affiliated Hospital of Integrated Traditional Chinese and Western Medicine, Nanjing University of Chinese Medicine, Nanjing, 210028 Jiangsu China

**Keywords:** Asperosaponin VI, Human mesenchymal stem cell, Differentiation, Nucleus pulposus-like cell, ERK1/2, smad2/3

## Abstract

**Background:**

The regeneration of nucleus pulposus (NP) cells is an effective method to prevent intervertebral disc degeneration (IVDD). In this study, we investigated the role of Asperosaponin VI (ASA VI), isolated from a traditional Chinese medicine (TCM), the root of *Dipsacus asper* Wall, in promoting human mesenchymal stem cell (HMSC) proliferation and differentiation into NP-like cells and explored the possible mechanism of action.

**Methods:**

The effects of ASA VI on HMSC viability and proliferation were determined by the XTT method and EDU staining. Then, Real-time qPCR, immunocytochemistry and immunofluorescence assays were used to measure the effect of ASA VI on the expression of extracellular matrix (ECM) components, such as COL2A1, aggrecan, SOX9, KRT19, PAX1, and glycosaminoglycans (GAGs), in NP cells. In addition, Western blot assay was used to measure the expression of p-ERK1/2 and p-smad2/3.

**Results:**

ASA VI was able to promote the proliferation and differentiation of HMSCs into NP-like cells, and the optimum concentration was 1 mg/L. Western blot assay indicated that the possible mechanism might be related to the activation of p-ERK1 / 2 and p-Smad2 / 3.

**Conclusions:**

ASA VI can promote the proliferation and differentiation of HMSCs into NP-like cells, which can potentially be used as a treatment for IVDD.

**Supplementary Information:**

The online version contains supplementary material available at 10.1186/s12906-020-03169-y.

## Introduction

Low back pain (LBP) is a common condition, and approximately 80% of the general population experiences it at least once in their lives [1]. Although there are many factors that can lead to LBP, intervertebral disc degeneration (IVDD) is considered a major factor [2, 3]. IVDD is related to age, load-bearing labor, trauma and heredity and leads to chronic LBP and an economic burden [4]. Treatment of IVDD remains a clinical challenge; conservative treatment alleviates only symptoms, whereas surgical treatment is associated with complications and structural damage [5]. There is no approved program for the treatment of degenerative intervertebral discs (IVDs). It is very important to find a treatment for the etiology of IVDD. Choi H et al. [6] found that a change in extracellular matrix (ECM) biosynthesis and decreases in the function and number of nucleus pulposus (NP) cells were the main factors associated with IVDD. Therefore, increasing ECM synthesis and promoting the regeneration of NP cells have become goals of treatment. Due to the poor regeneration of NP cells, cell-based therapy may be a promising treatment for IVDD [7]. Hyowon Choi et al. [8] suggested that stem cells can be used to replace old and damaged cells to replenish the disc structure. Moreover, a large number of experimental studies have confirmed that stem cell treatments can be effective and have great clinical potential [9, 10].

M Dominici et al. [11] proposed minimum criteria a human cell must meet to be defined as a human mesenchymal stem cell (HMSC): First, the cell must be plastic-adherent when maintained in standard culture conditions. Second, it must express CD105, CD73 and CD90 and lack expression of CD45, CD34, CD14 or CD11b, CD79a or CD19 and HLA-DR surface molecules. Third, the cell must be capable of differentiating into osteoblasts, adipocytes or chondroblasts in vitro.

At present, the expression levels of aggrecan, COL2A1 and SOX9 are commonly used to identify mesenchymal stem cell (MSC) acquisition of an NP phenotype [12–14]. Some scholars reported that ACAN is the most abundant proteoglycan in NP and that COL2A1 represents a substantial component of the NP matrix [15, 16]. Furthermore, KRT-19 has been identified as differentially expressed between NP and cartilage cells in bovine IVD [17, 18]. Minogue BM et al. [19] reported that PAX1 can be used as a novel NP marker and positive gene that it was more highly expressed in NP than in articular cartilage. In this study, the type II collagen, aggrecan, SOX9, KRT19 and PAX1 genes were used as phenotypic markers to identify NP cells.

There are many methods to promote the differentiation of stem cells into NP cells for the treatment of degenerative IVD, including growth factor intervention, coculture of stem cells and NP cells, hypoxia induction, stem cell seeding into three-dimensional scaffolds, and stress application [20–24]. However, traditional Chinese medicine (TCM) is rarely used to promote the differentiation of stem cells into NP-like cells to repair degenerative IVDs.

Asperosaponin VI (ASA VI), also known as Akebia Saponin D (ASD), is the main bioactive component of the TCM Radix Dipsaci. The Chinese Pharmacopoeia specifies a minimum ASA VI content as a *Dipsacus asper* Wall quality standard [25]. Dipsacus asper Wall, as an herbal medicine, has the effect of tonifying the liver and kidney and has a long history of safe use for strengthening the tendons and bones. Studies have found that Radix Dipsaci functions by inhibiting osteoclast differentiation, preventing osteoporosis and promoting fracture healing [26–28]. Numerous signaling pathways are regulated by ASA VI, including the PI3K/AKT, HIF-1a/VEGF, p38, ERK1/2 and smad2/3 pathways [29–32]. Previous studies have found that the ERK and Smad signaling pathways are involved in the proliferation and differentiation of stem cells [33–35]. In recent years, the ERK1/2 and smad2/3 signaling pathways have been found to regulate the differentiation of stem cells into NP-like cells and cartilage cell-like cells [36, 37]. In this study, it was hypothesized that ASA VI may promote HMSC differentiation into NP-like cells.

In this study, we investigated the effects of ASA VI on the proliferation and differentiation of HMSCs into NP-like cells and the possible mechanisms by evaluating the expression of p-ERK1/2 and p-smad2/3 at the protein level.

## Materials and Methods

### Materials

ASA VI was purchased from Chengdu Must Bio-Technology Co. Ltd. (purity> 99%, China). Mesenchymal stem cell medium (MSCm) (7501), fetal bovine serum (FBS, 7552) and Dulbecco’s phosphate-buffered saline (DPBS, 0303) were purchased from ScienCell (USA). DMEM/F12 was purchased from Gibco (21,041,025, USA). BeyoClick EdU-488 was purchased from Beyotime Institute of Bio-Technology Co. Ltd. (Beyotime, C0071S, China). ProtoScript II cDNA was purchased from NEB (m3003L, USA). DAPI (D9542), dimethylmethylene blue (DMMB, 341088), glycine (410225), glacial acetic acid (S7653) and bovine chondroitin 4-sulfate as standard (C9819) were purchased from Sigma-Aldrich (USA). Primary antibodies for β-catenin (ab179467) and paxillin 1 (PAX1, ab32084) were purchased from Abcam (USA). Aggrecan was purchased from Proteintech (13880–1-AP, USA), and smad2/3 (8685 T), p-smad2/3 (8828S), ERK1/2 (4695 T), and p-ERK1/2 (4370 T) were purchased from Cell Signaling (USA). Anti-rabbit secondary antibodies were purchased from Abcam (ab150077, USA).

### Cell culture

HMSCs were purchased from ScienCell (7500, USA). The cell line was cultured in MSCm supplemented with 10% FBS, 1% penicillin and 1% streptomycin at 37 °C in a humidified atmosphere of 5% CO_2_ in a T-75 flask for 48 h before the first medium change. Once the cells exceeded 80% confluence, they were passaged into T-75 flasks at a ratio of 1:3. HMSCs from the sixth passage were used in all experiments. For all subsequent experiments except the cell vitality and proliferation assays, the culture medium was replaced with DMEM/F12 supplemented with FBS (Invitrogen, 1,600,044, USA), dexamethasone (Sigma-Aldrich, D1756, USA), ascorbic acid-2-phosphate (Sigma-Aldrich, A4544, USA), L-proline (Sigma-Aldrich, P0380, USA), ITS Supplement (Cyagen Biosciences Company, 10,201, USA), and TGF-β1 (PeproTech, 96–100–21-10, USA).

### XTT assay

Cell **v**iability was evaluated by Cell Proliferation Kit (XTT) assays (Sigma-Aldrich, X4626, USA) according to the manufacturer’s instructions. In brief, HMSCs were seeded on 96-well plates (2 × 10^3^ cells/well) at 37 °C in a humidified atmosphere of 5% CO_2_ for 24 h. Next, the cells were treated with one of several concentrations of ASA VI (0, 0.01, 0.1, 1, 10, and 100 mg/L). The MSCm and ASA VI were changed every 48 h. After 1, 3 or 5 days, 50 μL of XTT working solution was added to each well, and the plates were cultured at 37 °C in a humidified atmosphere of 5% CO_2_ for 4 h. The absorbance was measured at 450 nm using a microplate reader (Bio-Tek, USA) after each addition of XTT working solution.

### Assessment of cellular proliferation

Cell proliferation was assessed by an EDU test using a BeyoClick EdU-488 cell proliferation kit combined with DAPI staining according to the manufacturer’s instructions. The HMSCs were plated on 96-well plates (2 × 10^3^ cells/well) at 37 °C in a humidified atmosphere of 5% CO_2_ for 24 h. Then, different doses of ASA VI (0, 0.01, 0.1, and 1 mg/L) were added to the wells, and the MSCm and ASA VI were changed every 48 h. Five days later, EDU reagent (0.5 μL, 50 μmol/L) was added to each well containing 200 μL of MSCm and incubated at 37 °C in a humidified atmosphere of 5% CO_2_ for 2 h. Then, the cells were fixed with 4% paraformaldehyde for 5 min. Next, 100 μL of penetrant was added to each well, and the cells were incubated for 5 min. Then, each well was supplemented with 100 μL of 1x EDU working fluid, and 30 min later, DAPI was used to stain the cell nuclei for 10 min [30]. The stained cells were observed under a fluorescence microscope (OLYMPUS, Japan).

### Quantitative real-time polymerase chain reaction (PCR)

The HMSCs were plated on 6-well plates (3 × 10^5^ cells/well). Then, the cells were treated with various concentrations of ASA VI (0, 0.01, 0.1, 1, 10, and 100 mg/L). The mixture was changed every 48 h. Total RNA was extracted from cells after 3 and 7 days using TRIzol reagent (INV, 15596026, USA) according to the manufacturer’s instructions. Then, cDNA was synthesized by applying a cDNA reverse transcription kit according to the manufacturer’s instructions. In brief, an 8-μL reaction mixture containing 1 μg of total RNA, 2 μL of oligo d(T)23VN (50 μM) and RNase-free dH_2_O was incubated at 65 °C for 5 min. Subsequently, 10 μL of ProtoScript II Reaction Mix (2X) and 2 μL of Proto-Script II Enzyme Mix (10X) were added to produce a final volume of 20 μL, and the mixture was incubated at 42 °C for 60 min. Finally, the mixture was incubated at 80 °C for 5 min. Gene expression was analyzed by quantitative real-time PCR (ABI Stepone Plus, USA). GAPDH was used to quantify the PCR products to confirm the use of equivalent RNA. Reactions were carried out in duplicate in a 96-well plate with a final volume of 20 μL. The PCR program included an initial enzyme activation stage at 95 °C for 10 min, followed by 40 cycles of 95 °C for 15 s and 60 °C for 60 s. Products were quantified using a melting curve analysis. The results were calculated using the 2^−ΔΔct^ method. The primers used in this study are shown in Table 1.

**Table 1:** Primers sequences used for RT-PCR analyses

Name		5→sequence→3	Product size	NCBI Reference Sequences(Ref Seq)
GAPDH	Sense	CCAGAACATCATCCCTGCCT	185	NM_00125679
	Antisense	CCTGCTTCACCACCTTCTTG		
COL2A1	Sense	TCCACGGAAGGCTCCCAGAA	141	NM_001844.5
	Antisense	CCTGCTATTGCCCTCTGCCC		
Aggrecan	Sense	CCTCTGGACAACCAGGTATTAG	97	NM_001135
	Antisense	CCAGATGTTTCTCCACTCAGAT		
SOX9	Sense	GAGCTGAGCAGCGACGTCAT	130	NM_000346.4
	Antisense	CGTAGCTGCCCGTGTAGGTG		
KRT19	Sense	GGAAGACACACTGGCAGAAA	112	NM_002276.5
	Antisense	CTCACTATCAGCTCGCACATC		
PAX1	Sense	CCGCTCGCTATGGAGCAGAC	204	NM_001257096.1
	Antisense	GGAGCCGGTCTCGTTGTAGC		

### Glycosaminoglycans (GAGs) assay

The HMSCs were plated on 6-well plates (3 × 10^5^ cells/well). After 24 h, cells were treated with the appropriate concentration of ASA VI (1 mg/L) or left untreated (control group). The medium was changed every 48 h. To investigate the effect of ASA VI on secreted ECM proteins, a DMMB assay was used to quantify the soluble GAGs in the cell culture medium. After 7 and 14 days, the cell culture medium were collected. A portion of the medium was mixed with DMMB dye and incubated with moderate agitation at room temperature for 30 min. Upon incubation, the solution was centrifuged to form a pellet of GAGs that bound to the dye. The pellet was washed in ice-cold acid-salt solution, centrifuged and resuspended in 10% SDS for the DMMB assay. The DMMB assay results were quantified at 525 nm using a microplate reader (Bio-Tek, USA). Absorbance was converted to GAG concentration using a calibration curve obtained using different concentrations of bovine chondroitin 4-sulfate as the standard. The GAGs were normalized using the total protein in the medium quantified at UV 280 nm with a microplate reader (Bio-Tek, USA) [38].

### Immunofluorescence staining

The HMSCs were plated on 96-well plates (2 × 10^3^ cells/well) at 37 °C in a humidified atmosphere of 5% CO_2_ for 24 h. Then, cells were treated with the appropriate concentration of ASA VI (1 mg/L) or left untreated (control cells). The medium was changed every 48 h. After 14 days, cells were fixed with 4% paraformaldehyde for 5 min. Next, each well was treated with 0.2% Triton X-100 in 1X PBS for 5 min at room temperature. Cells were then blocked with 5% blocking serum from specific species in 1X PBS at room temperature for 1 h. Subsequently, cells were incubated with primary antibody (1:50) diluted in antibody dilution buffer for 1 h at room temperature, followed by incubation with the corresponding fluorochrome-labeled secondary antibodies diluted in antibody dilution buffer (1:200) for 1 h at room temperature in the dark. Finally, DAPI was used to stain the cell nuclei for 10 min [39]. The stained cells were observed under a fluorescence microscope (OLYMPUS, Japan).

### Western blotting analysis

The HMSCs were plated on 6-well plates (3 × 10^5^ cells/well). After 24 h, the cells were treated with the appropriate concentration of ASA VI (1 mg/L) or left untreated (control cells). After 48 h, cells were washed with PBS and lysed with lysis buffer mixed with PMSF for 30 min on ice. Next, the cells were subjected to ultrasonic fragmentation (400 W; pulse duration, 15 s, pause duration, 15 s) for 10 min on an ice bath. After centrifuging for 10 min at 10,000 xg and 4 °C, the mixture was supplemented with loading buffer (5:1) and boiled in boiling water for 5 min. Protein samples were separated by 10% SDS-PAGE under 80 V for 30 min and 100 V for 90 min and then transferred to nitrocellulose membranes. The membranes were blocked with TBS buffer for 1 h at room temperature. The primary antibodies (rabbit polyclonal anti-ERK1/2, anti-phosphospecific ERK1/2, anti-smad2/3, anti-phosphospecific smad2/3, all at 1/500 dilution; rabbit monoclonal anti-β-actin, 1/5000 dilution) were added to the nitrocellulose membranes, which were incubated overnight at 4 °C. Subsequently, the membranes were washed three times for 5 min each time with TBS buffer and incubated with anti-rabbit secondary antibodies (1:5000) for 1 h at room temperature. The membranes were again washed three times for 5 min each time, and detection was performed using a dual-color infrared imaging system (Odyssey, LI-COR, USA).

### Statistical analysis

The results are expressed as the mean ± standard deviation. The statistical significance of group differences was determined using a one-way ANOVA or t-test in SPSS 19.0 statistical software. For each test, at least three independent parallel experiments were performed. *P* < 0.05 was considered to indicate statistical significance.

## Results

### Effect of ASA VI on HMSC proliferation

**Fig. 1:**
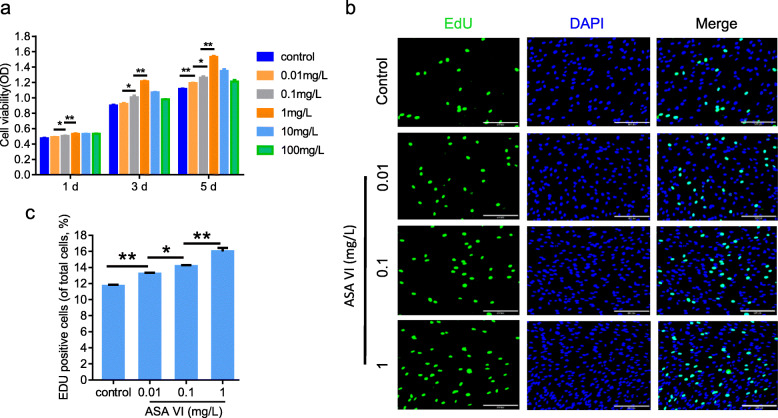
ASA VI promoted the proliferation of HMSCs. **a** Cell viability of HMSCs treated with different concentrations of ASA VI for 1, 3 or 5 days. **b** EDU staining of HMSCs after treatment with different concentrations of ASA VI (scale bar: 200 μm). **c** The relative number of HMSCs stained by EDU in each group. *p < 0.05, **p < 0.01

The effect of ASA VI on the proliferation of HMSCs was evaluated by XTT and EDU assays. Cell numbers were increased after incubation with different concentrations of ASA VI (0.01, 0.1, 1, 10, and 100 mg/L) for 1, 3 and 5 days. Cell proliferation peaked at 1 mg/mL (Fig. 1a), decreasing thereafter. Similar to the XTT results, the EDU assay results showed that the proliferation of HMSCs can be enhanced by ASA VI at doses up to 1 mg/mL (Fig. 1b and c).

### NP gene expression in cells under different concentrations of ASA VI

**Fig. 2:**
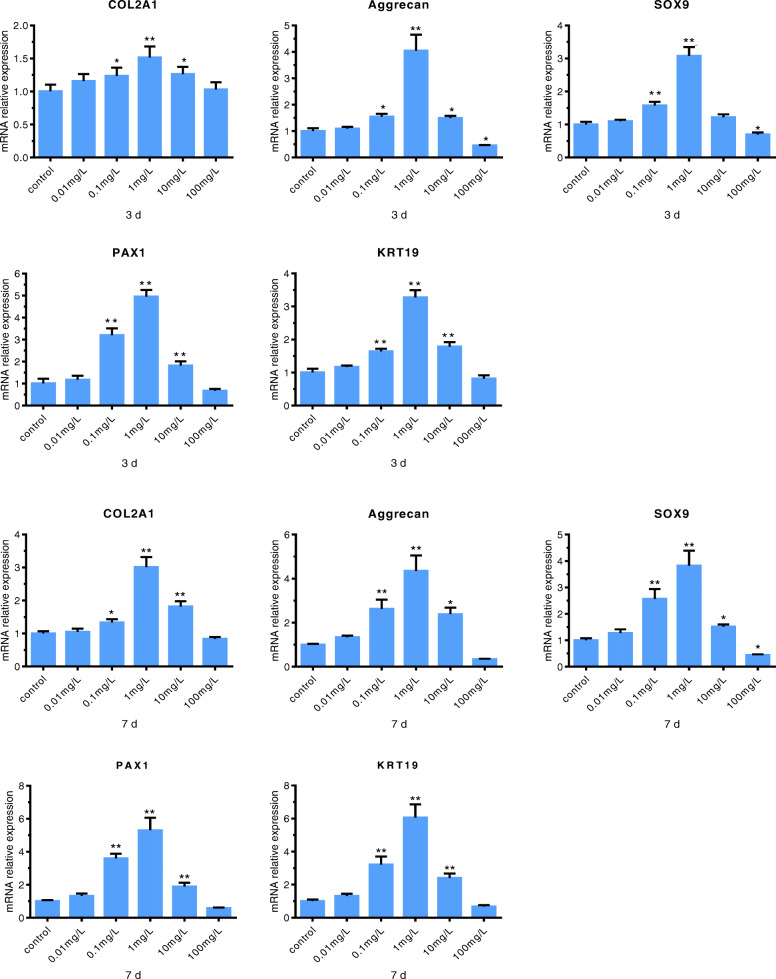
Effects of ASA VI concentration on gene expression. The expression levels of selected genes were assessed by RT-PCR after cells were treated with different concentrations of ASA VI for 3 or 7 days and normalized to the housekeeping gene, GAPDH. The control group was considered “1”. Compared with the control group, ASA VI upragulated the expression of the genes, and the optimal concentration was 1 mg/L .. *p < 0.05 vs controls, **p < 0.01 vs controls

To study the effects of different concentrations of ASA VI on the biosynthesis of HMSCs, ECM expression was analyzed by RT-PCR. Gene expression profiles were investigated after 3 and 7 days of ASA VI cultivation at 0 (control), 0.01, 0.1, 1, 10 and 100 mg/L. Figure 2 shows the relative gene expression of the NP markers (type II collagen, aggrecan, SOX9, KRT19, and PAX1) in the ASA VI and control groups. The results showed that ASA VI concentrations of 0.01, 0.1 and 1 mg/L ASA VI led to upregulated gene expression of the markers, which peaked at 1 mg/L. However, when the concentration of ASA VI increased to 10 mg/L and 100 mg/L, gene expression appeared to be inhibited.

These findings indicated that 1 mg/L was the optimal concentration of ASA VI for stimulating HMSC differentiation into NP-like cells. Thus, we adopted this concentration for the subsequent experiments.

### GAG expression under ASA VI

**Fig. 3:**
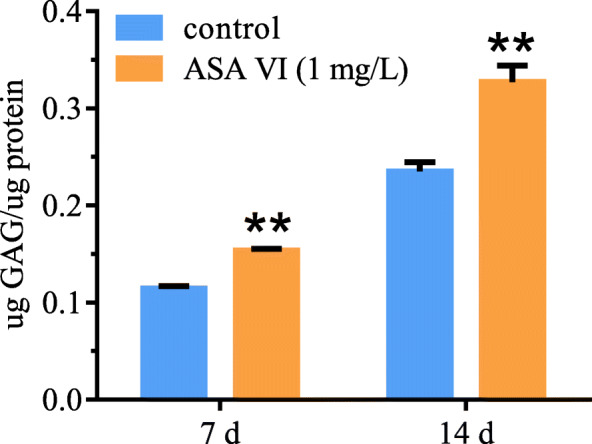
Effect of ASA VI concentration on the amount of GAGs in the culture medium. Treatment with ASA VI at 1 mg/L yielded the highest amount of secreted GAGs in the culture medium. **p < 0.01 vs controls

The levels of GAG expression were investigated after 7 and 14 days of ASA VI cultivation at 0 (control) or 1 mg/L. Figure 3 shows that the GAG contents in the cell supernatant significantly increased with time in both the experimental and control groups. The rate of increase in supernatant GAG content was higher in the experimental group than in the control group at 7 and 14 days.

### ASA VI accelerated aggrecan and PAX1 deposition

**Fig. 4:**
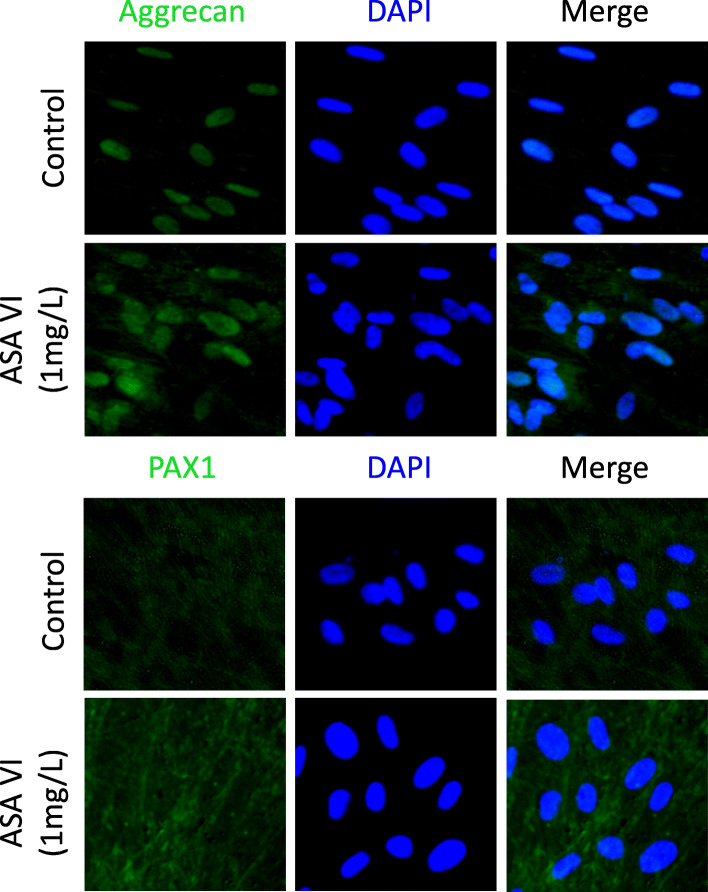
The expression levels of aggrecan and PAX1 genes were verified by immunostaining. The immunofluorescence signals were stronger (green staining) in the experimentally treated cells than in the controls (scale bar: 200 μm)

Aggrecan and PAX1 immunofluorescence staining revealed stronger green staining in the ASA VI-treated groups (1 mg/L) compared with the control groups after 14 days of culture, which suggests a greater abundance of regenerated aggrecan and greater PAX1 deposition in the ASA VI-treated groups (Fig. 4).

### ASA VI upregulated p-ERK1/2 and p-Smad2/3 expression

**Fig. 5:**
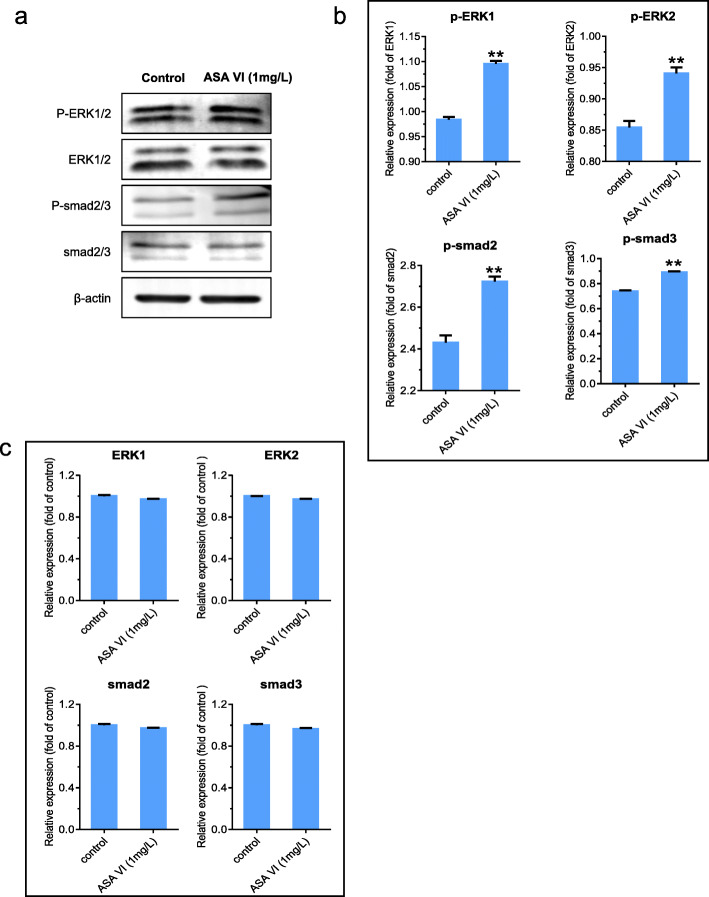
The effects of ASA VI on the protein expression levels of p-ERK1/2, p-smad2/3, ERK1/2 and smad2/3. Western blot analysis of p-ERK1/2、p-Smad2/3 、ERK1/2 and smad2/3 expression levels after treatment with ASA VI for 48 h. p-ERK1/2 and p-Smad2/3 levels were normalized to those of ERK1/2 and smad2/3(Fig. 5b) and the protain levels of ERK1/2 and smad2/3 were folded of control (Fig. 5c). ** *p* < 0.01 vs controls

To investigate the mechanism by which ASA VI promotes the differentiation of HMSCs into NP-like cells, we explored the effects of ASA VI on p-smad2/3 、p-ERK1/2 、ERK1/2 and smad2/3 expression using Western blotting. The results indicated that ASA VI (1 mg/L) could upregulate the protein expression of both p-smad2/3 and P-ERK1/2 relative to that in the control group (Fig. 5b). However, the protein levels of ERK1/2 and smad2/3 had no significant difference compared to the control group (Fig. 5c).

## Discussion

Compared with traditional therapies, biotherapy may be more beneficial for relieving pain, repairing degenerative NP and restoring the biomechanical function of IVD [40]. Therefore, inducing stem cells to differentiate into NP-like cells has become the focus of biotherapy for IVDD, and identifying effective methods to do so is a key challenge in IVDD treatment [41]. Promoting the phenotypic differentiation of stem cells into NP cells is the basis of NP regeneration.

By comparing the phenotypes of cartilage cells and NP cells, we found that COL2A1, aggrecan and SOX9 expression was shared by NP cells and cartilage cells [17, 42]. However, NP cells are significantly different from chondrocytes in composition and biological function. Thus, to ensure the accumulation of an appropriate amount of ECM, it is necessary to identify the phenotypes of differentiated cells. KRT19, as a specific marker of human chordae, was used to identify positive markers in NP cells [43]. Thorpe AA et al. [44] found that KRT19 can be used as a unique marker for the identification of NP cells. PAX1 is involved in the regulation of IVD formation in the embryonic stage and has been identified in human NP cells. Moreover, it is widely used as a new phenotypic marker for the identification of NP cells in the study of stem cell differentiation into NP cells [44]. Therefore, we believed that genes such as COL2A1, aggrecan, SOX9, KRT19 and PAX1 can be used as genetic markers to identify NP cells.

As the main bioactive component of Radix Dipsaci, ASA VI has the effects of regulating intestinal microflora, preventing atherosclerosis, resisting inflammation, reducing cortisol, promoting angiogenesis, and promoting fracture and wound healing [28, 45–48]. In this study, We found that at a concentration of 1 mg/l, ASA VI not only promoted the expression of the NP cell genes COL2A1, aggrecan, SOX9 and matrix deposition, but aso promoted the expression of the NP marker and positive genes KRT19 and PAX1.Thus, we can conclude that ASA VI may be more conducive to promoting hMSC differentiation into NP-like cell than other induction methods for the treatment of IVDD. At the same time, ASA VI also showed the ability to stimulate the proliferation of HMSCs. However, proliferation and differentiation are inversely related phenomena. In the differentiation experiment, we introduced TGF-β1. TGF-β1 has diverse biological functions in multiple cellular processes such as regulating proliferation and differentiation of stem cells [49, 50]. Hence, we thought that TGF-β1 might regulate the proliferation and differentiation of HMSCs. Meanwhile, we found that ASA VI increased the expression of p-ERK1/2 and p-smad2/3. It has been reported that ERK1/2 and smad2/3 pathways can regulate the proliferation and differentiation of stem cells [33, 51]. Thus, we suggested that ASA VI might promote the proliferation and differentiation of HMSCs into NP-like cells by activating ERK1/2 and smad2/3 pathways.

Signal transduction mediated by members of the MAPK family involves a multistep kinase cascade. ERK1/2 is a major kinase among MAPKs, which can activate important cellular processes, including key transcriptional and phenotypic differentiation programs [52, 53]. ERK1/2 plays an important role in stimulating cell proliferation and differentiation, especially in promoting the differentiation of stem cells into NP-like cells [54, 55]. The smad family plays key roles in the transmission of TGF-β signals from cell surface receptors to the nucleus. TGF-β has been identified as a new signal agonist. Yang J et al. [51] reported that TGF-β critically regulates cell differentiation through the main signal transducer smad2/3 in HESCs. Several studies have indicated that TGF-β can promote the differentiation of stem cells into NP-like cells [56, 57]. Interestingly, in our study, ASA VI treatment simultaneously activated the ERK1/2 and smad2/3 pathways. We believe that the activation of both pathways is not contradictory. The two pathways might be related to each other in terms of NP-like cell differentiation. Li J et al. [52] reported that ERK1/2 and smad2/3 signals intersected in the regulation of cartilage differentiation. Furthermore, Hough et al. [58] found that the phosphorylation of ERK can regulate Smad signaling. However, the association between ERK1/2 signaling and smad2/3 signaling remains unclear. Moreover, although we have demonstrated that ASA VI can promote the differentiation of HMSCs into NP-like cells in vitro, how this compound regulates several signaling pathways remains to be elucidated.

## Conclusions

We found that ASA VI promoted the proliferation and differentiation of HMSCs into NP-like cells probably by activating the ERK1/2 and smad2/3 signaling pathways. At the same time, TGF-β1 might be as a possible cause behind the synergistic effect in regulating the proliferation and differentiation of HMSCs into NP-like cells. Our research increases our understanding of the potential mechanism by which ASA VI promotes the differentiation of HMSCs into NP-like cells and suggests that ASA VI has therapeutic potential in the treatment of IVDD with stem cells.

## Supplementary Information

**Additional file 1.** Uncropped original Figures of Western blot of Figure 5.

## Data Availability

The datasets used and/or analyzed during the current study are available from the corresponding author on reasonable request.

## Abbreviations

IVD: Intervertebral disc
IVDD: Intervertebral disc degeneration
ASA VI: ASPEROSAPONIN VI
TCM: Traditional Chinese medicine
HMSC: Human mesenchymal stem cell
XTT: 2,3-bis-(2-methoxy-4-nitro-5-sulfophenyl)-2 h-tetrazolium-5-carboxanilide
EDU: 5-ethynyl-2-deoxyuridine
COL2A1: Type II collagen
SOX9: SRY-related high mobility group-box gene 9
PAX1: Paxillin 1
KRT19: Cytokeratin 19
GAGs: Glycosaminoglycans
ERK: Extracellular regulated protein kinases
Smad: Small mothers against decapentaplegic
LBP: Low back pain
ECM: Extracellular matrix
PI3K: Phosphatidylinositol 3 kinase
AKT: Protein kinase B
HIF-1a: Hypoxia-inducible factor 1-alpha
VEGF: Vascular endothelial growth factor
p38: Protein 38
MSCm: Mesenchymal stem cell medium
FBS: Fetal bovine serum
PBS: Phosphate-buffered saline
DPBS: Dulbecco’s phosphate buffered saline
DMEM: Dulbecco’s modification of Eagle’s medium
DAPI: 4,6-diamino-2-phenyl indole
EDTA: Ethylene diamine tetraacetic acid
TGF-β1: Transforming growth factor beta 1
PCR: Polymerase chain reaction
RNA: Ribonucleic acid
DNA: Deoxyribonucleic acid
GAPDH: Glyceraldehyde-3-phosphate dehydrogenase
DMMB: Dimethylmethylene blue assay
PMSF: Phenylmethanesulfonyl fluoride
SDS: Sodium dodecyl sulfate
SDS-PAGE: Sodium dodecyl sulfate-polyacrylamide gel electrophoresis
hESCs: Human embryonic stem cells
