# Supplementary figures and images for: In the presence of TGF-β1, Asperosaponin VI promotes human mesenchymal stem cell differentiation into nucleus pulposus like- cells
Source: BMC Complement Med Ther. 2021 Jan 14;21:32. doi: 10.1186/s12906-020-03169-y (PMC7807821; doi:10.1186/s12906-020-03169-y)

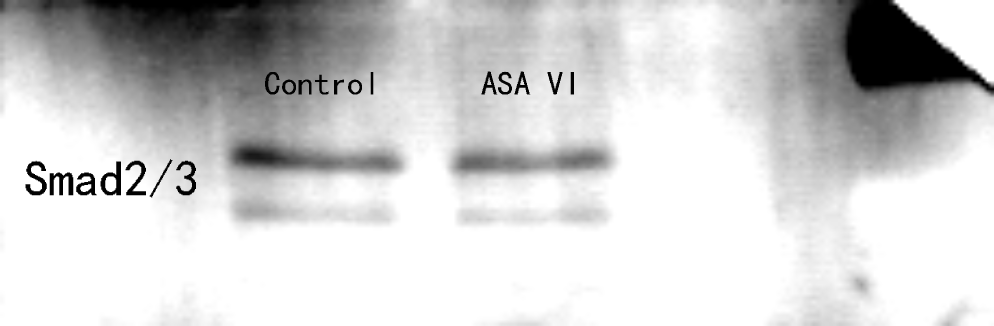

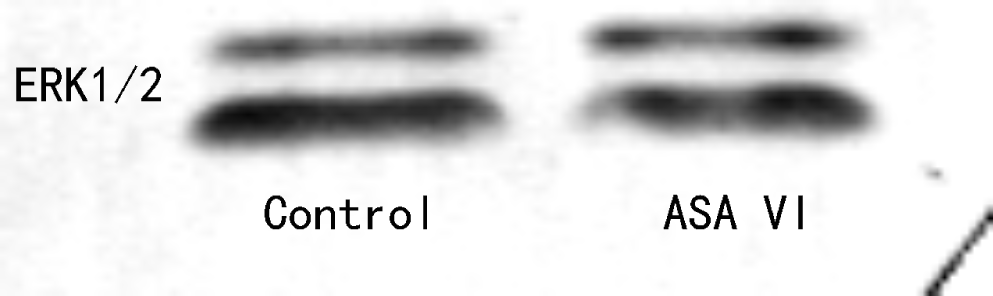

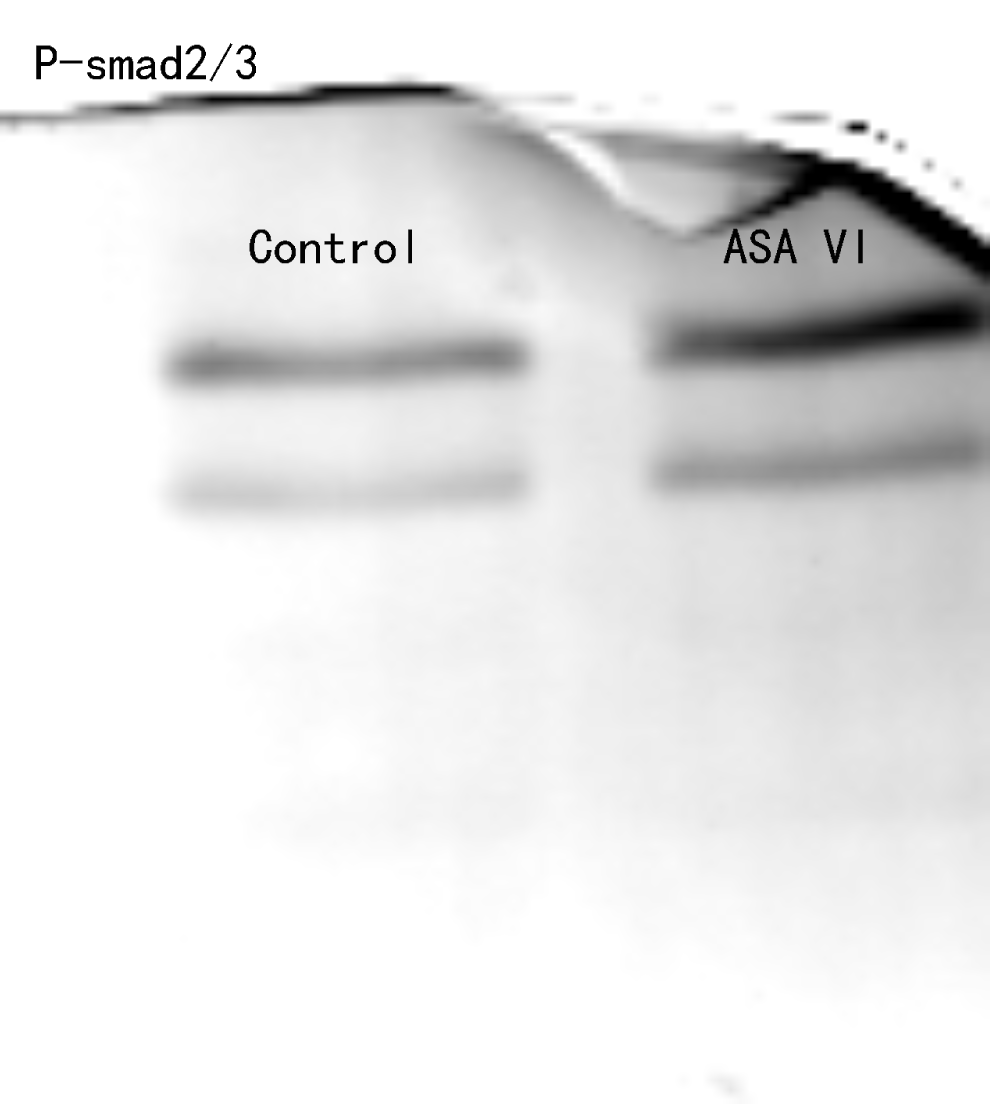

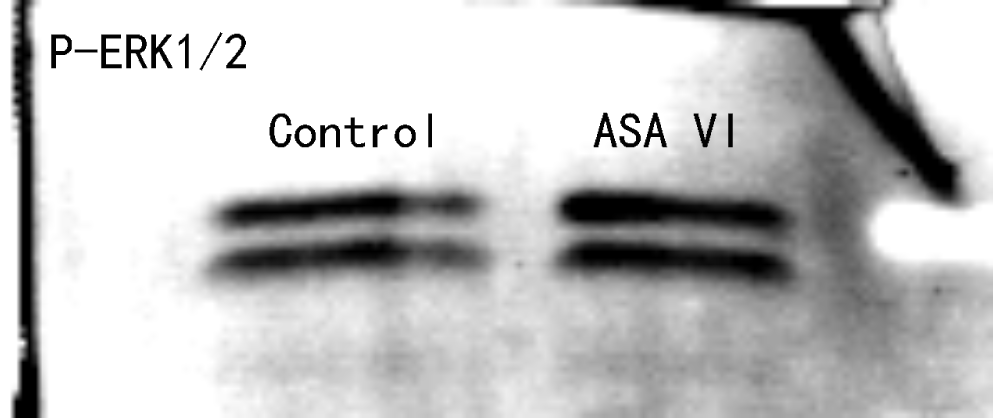

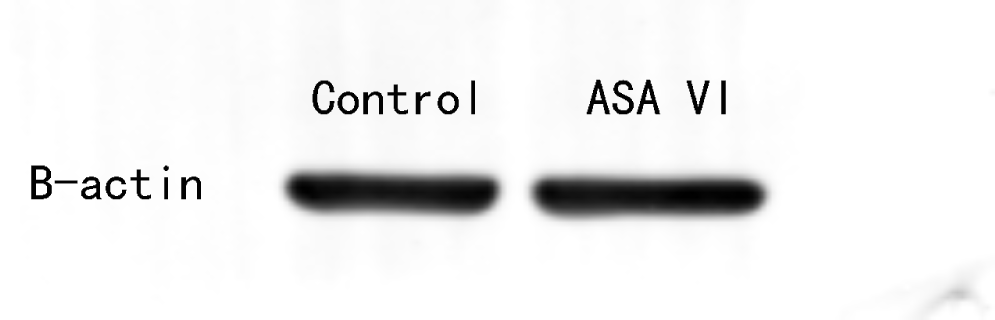

Supplement: Supplementary file 1 — Additional file 1. Uncropped original Figures of Western blot of Figure 5. [file 12906_2020_3169_MOESM1_ESM.docx]
